# Greek Physicians' Perceptions on Generic Drugs in the Era of Austerity

**DOI:** 10.1155/2015/251792

**Published:** 2015-09-17

**Authors:** Georgios Labiris, Michael Fanariotis, Catherine Kastanioti, Georgios Alexias, Adonis Protopapas, Theodoros Karampitsakos, Dimitris Niakas

**Affiliations:** ^1^Medical School, Democritus University Medical Campus, 1st Building, 68100 Alexandroupolis, Greece; ^2^Faculty of Social Sciences, Hellenic Open University, 26335 Patras, Greece

## Abstract

*Purpose. *To assess the beliefs and preferences of Greek physicians, regarding generic drugs, in the years of financial crisis. *Setting. *Multicentered, nationwide survey. *Material and Methods. *A custom questionnaire based on former similar studies was developed and administered to Greek physicians. The variable “perception on generics” was constructed after an exploratory study and the instrument was validated by conventional and Rasch analysis methods. 22 items formed 5 subscales that constructed the variable in question. *Results. *908 physicians successfully participated in the study (response rate: 80%). Mean total scores to the instrument were 60.63 ± 12.12 for men and significantly less (58.24 ± 11.73) for women (*p *= 0.04). Greek physicians were not persuaded on the potential economic gain (45.79 ± 10.53); moreover they identified that Greek authorities cannot address the increased pharmacovigilance mandates. Physicians working in Athens and those working in surgical units demonstrated significantly worse scores than their colleagues from the rest of Greece and those working in Internal Medicine wards (*p* = 0.03).  *Conclusion*. Our results suggest an overall poor acceptance of the national initiative on generic drugs by Greek physicians. This trial is registered with Clinicaltrials.gov identifier: NCT01855802.

## 1. Introduction

Generic medicines have always been considered as an essential and integral part of the healthcare delivery systems across Europe [1]. Further to the direct savings, generics have stimulated the competition within the pharmaceutical sector to the benefit of the beneficiaries. However, generic market penetrance is far from uniform in the European Union (EU) for a series of reasons; among them are lack of coherent policies, variations in reimbursement systems, and major differences in the overall management of member-countries National Healthcare Systems (NHS) [2, 3]. Moreover, major socioeconomic discrepancies, especially between the core-European and the southern countries resulted in a segmented market that does not foster common pan-European initiatives [4, 5].

Greece has traditionally been among the countries with the highest pharmaceutical healthcare expenditures [6, 7] and the lowest generics market shares [7] primarily due to an overall poor regulation of the local pharmaceutical market which suffers from frequent periods of shortages and oversupplies of numerous drugs. Recently, due to the struggling economy and the deep recession, Greek NHS faced the pressing obligation of cost reduction in order to meet with the troika directives [8]. Among the initial cost-saving measures adopted in the Greek health system were the promotion of the generic consumption against original drugs with the introduction of a substitution policy, patient copayments, and a media-based strategy to reverse prescribing habits [9–11].

In fact, physicians' attitudes towards generics have been of major concern to researchers and policy makers since they directly predict the efficacy of any generic-promoting policy [12–17]. However, a thorough review of the international literature returned only one relevant study from Greece that was conducted before the economic crisis and used a custom, nonvalidated questionnaire [18]. Within this context, primary objective of the study was the assessment of Greek physicians' perceptions regarding generics in the local Healthcare Sector and exploring potential correlations with their specific medicine-based and demographic characteristics.

## 2. Materials and Methods

### 2.1. Setting

This was a multicentred, countrywide survey. Physicians from NHS Hospitals, Healthcare Centres, and private healthcare facilities in different locations were approached. The institutional review boards of the Democritus University of Thrace and the Hellenic Open University approved the protocol, written consent was obtained from all participants, and the survey was conducted during the period between February and April 2014.

### 2.2. Instrument Development

For the sake of the study, a survey instrument (questionnaire) was developed after conducting a systematic review of the literature pertinent to the subject in question. The search terms that were used in “Medline” search engine were combinations of the keywords “generics,” “physician,” and “perceptions,” in the title, in the keywords, and/or in the abstract. The timeframe of the search was up to 2013. The review of the literature resulted in 18 relevant publications, of which two were excluded since they were written in non-English languages (French and Spanish). The rest provided the necessary framework for the construction of the questionnaire. Following the literature review, an exploratory interview study was designed to create the baseline for the questionnaire development. A panel consisting of four clinical professors and a psychologist was recruited for the exploratory study. A number of items covering physicians' perceptions on the generics were summarized and written as interview questions. Individual interviews with 10 physicians who had no previous contact with any of the members of the panel took place. The interviews were analyzed and the findings served as the basis for identifying the variables of interest that would be operationalized in specific questions to be used in our instrument. As a result, further to the demographic information, the questionnaire elicited information by means of 5-point Likert scales on the following subscales (generics instrument (core questionnaire) is as below): (a) physicians' fundamental (core) perceptions regarding generics [FP] (items: 1–4), (b) physicians' perceptions on generic use in advanced or life-threatening states [LTP] (items 11-12, 14-15), (c) physicians' perceptions on the overall pharmacovigilance process in Greece [PP] (items 6–8, 21), (d) physicians' perceptions on the overall economic gain from generics [EGP] (items 5, 17–19, and 22–25), and (e) physicians' perceptions regarding generics impact on their professional relationship with their patients [PRP] (items 16, 20). Three more direct questions were addressed to the participants that attempted to explore their point of view on the automatic substitution measure that was introduced in the Greek pharmaceutical market (items 9-10, 13). All physicians were approached by a computer-based randomization selection program and responded to the instrument in the presence of an independent researcher, with no direct involvement in the study. Proxy responses and missing values were not allowed.

1. Generics and originals have the exact same reactive agent.
  - Strongly disagree.
  - Somewhat disagree.
  - Neither agree nor disagree.
  - Somewhat agree.
  - Strongly agree.
2. Generics and originals are therapeutically equivalent.
  - Strongly disagree.
  - Somewhat disagree.
  - Neither agree nor disagree.
  - Somewhat agree.
  - Strongly agree.
3. Generics and originals have the exact same safety profile.
  - Strongly disagree.
  - Somewhat disagree.
  - Neither agree nor disagree.
  - Somewhat agree.
  - Strongly agree.
4. Generics and originals have the exact same manufacturing standards.
  - Strongly disagree.
  - Somewhat disagree.
  - Neither agree nor disagree.
  - Somewhat agree.
  - Strongly agree.
5. In the majority of cases, price difference between generics and originals is so great that I feel pressured to prescribe generic substitutes.
  - Strongly disagree.
  - Somewhat disagree.
  - Neither agree nor disagree.
  - Somewhat agree.
  - Strongly agree.
6. Greek drug regulatory authority is capable of addressing any misconduct in the generics market (i.e., manufacturing, marketing).
  - Strongly disagree.
  - Somewhat disagree.
  - Neither agree nor disagree.
  - Somewhat agree.
  - Strongly agree.
7. Greek drug regulatory authority is capable of identifying and withdrawing any generics with suboptimal efficacy and/or safety.
  - Strongly disagree.
  - Somewhat disagree.
  - Neither agree nor disagree.
  - Somewhat agree.
  - Strongly agree.
8. In case of potential incompetence by the Greek drug authority, EU drug authorities are capable of identifying and addressing any misconduct in the generic market in Greece.
  - Strongly disagree.
  - Somewhat disagree.
  - Neither agree nor disagree.
  - Somewhat agree.
  - Strongly agree.
9. I support automatic substitution of original drugs by generics (i.e., by pharmacists) as a policy measure by Greek authorities.
  - Strongly disagree.
  - Somewhat disagree.
  - Neither agree nor disagree.
  - Somewhat agree.
  - Strongly agree.
10. Generic substitution should be only done by qualified physicians.
  - Strongly disagree.
  - Somewhat disagree.
  - Neither agree nor disagree.
  - Somewhat agree.
  - Strongly agree.
11. Generic substitution should not be performed in life-threatening diseases.
  - Strongly disagree.
  - Somewhat disagree.
  - Neither agree nor disagree.
  - Somewhat agree.
  - Strongly agree.
12. Generic substitution should not be performed in the advanced stages of any disease.
  - Strongly disagree.
  - Somewhat disagree.
  - Neither agree nor disagree.
  - Somewhat agree.
  - Strongly agree.
13. Corresponding medical colleges (e.g., College of Surgeons) should address specific guidance on generics according to the specific disease.
  - Strongly disagree.
  - Somewhat disagree.
  - Neither agree nor disagree.
  - Somewhat agree.
  - Strongly agree.
14. Generic substitution should not be performed in cases of imminent irreversible functional damage.
  - Strongly disagree.
  - Somewhat disagree.
  - Neither agree nor disagree.
  - Somewhat agree.
  - Strongly agree.
15. Generic substitution will facilitate my patients' compliance.
  - Strongly disagree.
  - Somewhat disagree.
  - Neither agree nor disagree.
  - Somewhat agree.
  - Strongly agree.
16. Generic substitution will improve my patients' confidence in my praxis.
  - Strongly disagree.
  - Somewhat disagree.
  - Neither agree nor disagree.
  - Somewhat agree.
  - Strongly agree.
17. Generic substitution will contribute to the overall cost-effective management of the corresponding disease.
  - Strongly disagree.
  - Somewhat disagree.
  - Neither agree nor disagree.
  - Somewhat agree.
  - Strongly agree.
18. Generic substitution will reduce illegal promotion practices from drug manufacturer companies.
  - Strongly disagree.
  - Somewhat disagree.
  - Neither agree nor disagree.
  - Somewhat agree.
  - Strongly agree.
19. Generic substitution will contribute to the overall cost-saving management of the corresponding disease.
  - Strongly disagree.
  - Somewhat disagree.
  - Neither agree nor disagree.
  - Somewhat agree.
  - Strongly agree.
20. Generic substitution will reduce my professional authority.
  - Strongly disagree.
  - Somewhat disagree.
  - Neither agree nor disagree.
  - Somewhat agree.
  - Strongly agree.
21. Generics that are manufactured in developing countries (like Pakistan or India) should be banned from the Greek market.
  - Strongly disagree.
  - Somewhat disagree.
  - Neither agree nor disagree.
  - Somewhat agree.
  - Strongly agree.
22. I feel pressured to request for additional examinations (e.g., X-rays, lab tests) when prescribing generics.
  - Strongly disagree.
  - Somewhat disagree.
  - Neither agree nor disagree.
  - Somewhat agree.
  - Strongly agree.
23. I feel pressured to request more frequent follow-up visits when prescribing generics.
  - Strongly disagree.
  - Somewhat disagree.
  - Neither agree nor disagree.
  - Somewhat agree.
  - Strongly agree.
24. Generics will discriminate my patients according to their out-of-pocket capability to purchase originals.
  - Strongly disagree.
  - Somewhat disagree.
  - Neither agree nor disagree.
  - Somewhat agree.
  - Strongly agree.
25. I believe that the average price of generics in Greece is higher than the rest of EU countries.
  - Strongly disagree.
  - Somewhat disagree.
  - Neither agree nor disagree.
  - Somewhat agree.
  - Strongly agree.

### 2.3. Validation Methods

According to item response theory, physician's perception regarding generics (either positive, neutral, or negative) is a latent variable that can be inferred from the person's report. By means of an item response model, it is possible to estimate the value of the latent variable on an interval scale from the item scores that form an ordinal scale. In brief, the application of item response theory in our study begins with the theoretical construction of the variable “perception on generics.” Each physician has a unique “perception on generics.” Depending on the physician's perceptions, certain questions will contribute to the development of the variable more than others. Taking into account the Rasch model assumptions, an estimate of “perception on generics” is possible on an interval scale. These assumptions are as follows: (a) persons recruited for the validation of the model differ on the perceptions on generics use, (b) responses to the items depend only on their perceptions on generics, and (c), responses are probabilistic and conditional on the person's perceptions on generics. Therefore, if an item is not sensitive enough for the latent variable, it will appear as noisy when evaluating the fit of data to the model. Similarly, if the physician's response is strongly influenced by reasons unrelated to generic use, that person's response pattern will be identified as outlying relative to the expectations of the model.

### 2.4. Statistical Analysis

Validation of the questionnaire was performed by estimation of the Cronbach alpha and by means of Rasch analysis. Total and subscale scores from all participants were summed for comparisons. Mean scores and standard deviations were presented for group comparisons. Normality of data was assessed with Kolmogorov-Smirnov testing, and parametric or nonparametric tests were applied, accordingly. Level of statistical significance was set at 0.05. Statistical analysis was performed using the Statistical Package for Social Sciences (SPSS, version 17.0).

## 3. Results

1140 physicians were approached for the sake of the study, and 908 of them (568 men, 340 women) with mean age 43.2 ± 10.2 years (youngest: 26, oldest: 67 years) responded successfully to the questionnaire (response rate: 80%). 56% were consultants at NHS or University Units, 35% were residents, and 9% worked in the private sector. The majority of the participants (30%) served in Internal Medicine and 35% were located in Athens. All demographic details are presented in Table 1.

### 3.1. Validation of the Instrument

**Figure 1:**
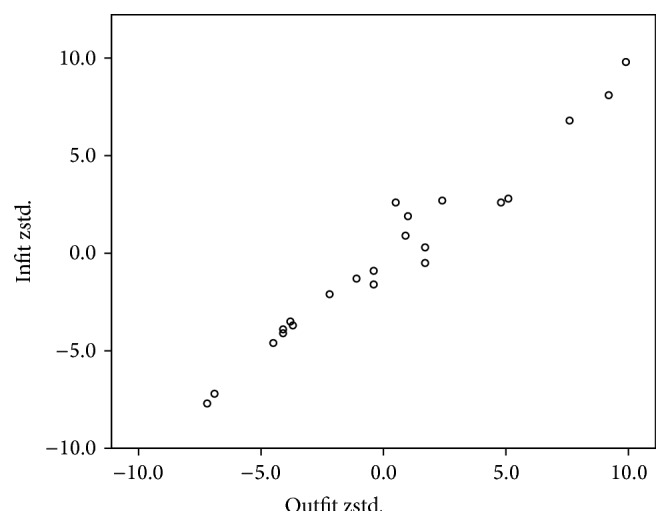
Rasch analysis. Infit and outfit statistics.

**Figure 2:**
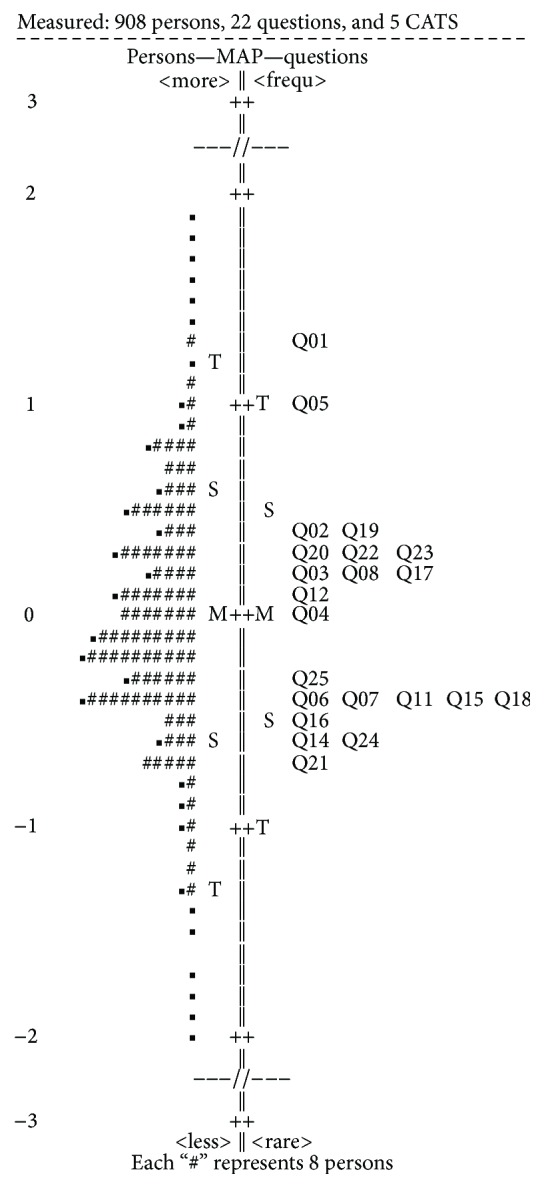
Rasch analysis. Item-person targeting.

Cronbach's alpha results are presented in Table 2. Overall the questionnaire demonstrated sufficient reliability, with the exception of the PRP subscale (*α* = 0.447). Regarding validity, all items passed the convergent and discriminant validity tests. On the other hand, the normalized item fit statistics are presented in Table 3. It is known that the expected values are 0, with a tolerance of ±2 deviation units. Positive values indicate that response residuals exceed the expectations of the model, while negative values are less than the expectations of the model. As presented in Figure 1, which illustrates the infit and outfit values, none of the items fell outside the tolerance box. Moreover, Figure 2 illustrates sufficient item-person targeting. Further to the fit statistics, principal component analysis of the residuals indicated average unidimensionality (principal component explained 58% of the variance).

### 3.2. Study Outcomes

Total and subscale scores are presented in Table 4. Higher scores reflect a more positive attitude towards generics. Maximal score is 100 for total and subscale scores. Mean total scores were 60.63 ± 12.12 for men and significantly less (58.24 ± 11.73) for women (*p* = 0.04). Worse scores were identified in the EGP subscale (45.79 ± 10.53), followed by the PP and LTP subscales. Best scores were detected in the FP subscale (68.65 ± 19.21). Physicians working in Athens demonstrated significantly worse scores than their colleagues from the rest of Greece (*p* = 0.03), as well as NHS physicians in comparison to those working in private units (*p* = 0.01). The rest of the demographic dimensions exerted no significant impact on the total score.

Regarding subscale comparisons, men presented significantly better scores at the FP (*p* = 0.01) and LTP (*p* < 0.01) subscales; consultants at the LTP subscale (*p* < 0.01); age (i.e., older physicians) at the PRP (*p* = 0.02); Internal Medicine Discipline (versus Surgical and Intensive Medicines ones) at the LTP subscale (*p* = 0.01) (detailed results not shown). On the other hand, 85% were against the automatic substitution process by the pharmacists, and 61% considered that generic substitution would discriminate their patients according to their out-of-pocket capability to purchase original drugs.

## 4. Discussion

The introduction of a successful initiative towards generic consumption requires a series of prerequisites; among them is the knowledge of the physicians' attitudes [13–22]. This is important because prescribers' perceptions predict the overall efficacy of the policy measures and prevent potential conflicts between care providers and the NHS authorities.

Contrary to the majority of former surveys that used custom, nonvalidated questionnaires, among the objectives of our study was the development of a validated instrument that would enable the construction of the variable “perception on generics” on an interval scale. That was essential in the study design, since we wanted to quantify the variable; that is, higher values indicated a more positive attitude towards generics, while lower values indicated a negative attitude. Therefore, direct comparisons with future studies with similar methods would be possible, rather than indirect referrals to study results. The instrument that we used in our study quantifies the variable “perception on generics” using five subscales, comprising 22 items. Regarding the validation process, Cronbach's alpha suggested adequate reliability with all items passing convergent and discriminant validity tests. Moreover, Rasch analysis indicated no misfitting items and adequate unidimensionality.

Regarding the results of our survey, total instrument scores suggested that our participants presented an average attitude on generics. Worse scores were identified in the EGP and the PP subscales, followed by the LTP subscale. These average scores suggest the following: (a) Greek physicians are not convinced about the potential overall economic gain from generics, (b) they are not convinced that NHS authorities can address the increased pharmacovigilance mandates that generic substitution requires, and (c) generics are most likely to be associated with suboptimal therapeutic outcomes and therefore they should not be used in life-threatening states or diseases with imminent irreversible damage to the patient's health.

The aforementioned results suggest that the national campaign to reverse prescribing patterns in Greece did not persuade Greek physicians. To our knowledge, poor results were inevitable since generic substitution was the flagship of an overall cost-saving policy in the Greek care delivery system that (a) excluded doctors with the introduction of the automatic substitution system at the pharmacy level, (b) accused both doctors and pharmaceutical companies for illegal practices, (c) was accompanied by shortages in the availability of generic drugs due to sector's challenged sustainability (either local manufacturers or importers) by the economic crisis, and (d) was associated with significant back-and-forths in economic objectives and relevant implementation strategies.

Moreover, the struggling Greek NHS, with all potential revenues generated by generics transferred to cover the national debt instead of being invested to the system and the pharmacovigilance service, further contributed to the negative attitude towards the initiative. On the other hand, well-known issues regarding generics and the automatic substitution process, among others, the reduced patient's compliance [23], the increased incidence of allergic reactions [24], and the negative attitude from beneficiaries [25, 26], were not addressed by the national campaign. Within this context, 85% of Greek physicians were against the automatic substitution process; moreover, 61% considered that it would discriminate their patients according to their out-of-pocket ability to purchase the original drug (that they had actually prescribed).

Therefore, Greek physicians presented even worse attitudes than their colleagues from other countries [15, 17]; although they identified that generics are supposed to have the same reactive agent and similar manufacturing standards, safety profile, and bioequivalence, they considered that they are, more often, associated with either suboptimal therapeutic outcome and/or increased adverse effects especially for those generics that are manufactured in developing countries [20]. Negative attitude on generics was more prominent in those physicians working at the major NHS Units in Athens and those serving at surgical disciplines. All of the above suggest an overall lack of confidence towards generics especially when they are supposed to be used in major NHS Units and especially in surgical patients who require a prompt pharmacologic response.

It becomes obvious that the strength of this study was the number of participants, their nationwide distribution, and the use of a validated instrument that allows the construction and quantification of the variable “perception on generics.” Among the limitations of the study was that the sample was not evenly stratified and some subscales of the questionnaire returned average reliability.

## 5. Conclusion

Our results indicate an overall poor acceptance of the national initiative for generic use and the automatic substitution process from the Greek physicians.

## Figures and Tables

**Table 1:** Demographics.

Age	43.2 (±10.2)
Sex	
Men	568
Women	340
Specialist/resident	
Specialist	596
Resident	312
Private/state	
Private	84
State	824
Years as a specialist	
1-2 years	108
3–5 years	166
5–10 years	82
10–15 years	84
15–20 years	84
>20 years	72
Resident	312
Medical discipline	
Emergency Medicine	194
General Practitioner	148
Laboratory	44
Internal Medicine	256
Paediatrics	14
Surgical	252
Location of praxis	
Rest of Greece	564
Athens	344

**Table 2:** Reliability analysis.

Subscale	Number of items	Cronbach's alpha
Fundamentals	4	0.841
State Audit	4	0.639
Empathy	4	0.558
Fiscal	6	0.647
Interaction	2	0.447
Resources util.	2	0.871
All	22	0.874

**Table 3:** Normalized item fit statistics.

Item number	*ρ*	Standard error	Infit zstd.	Outfit zstd.
18	0.72	0.05	9.8	9.9
9	0.36	0.04	8.1	9.2
10	−0.09	0.04	6.8	7.6
5	−0.99	0.05	2.8	5.1
22	0.32	0.04	2.6	4.8
1	−1.26	0.06	2.6	0.5
15	0.38	0.04	2.7	2.4
11	0.59	0.05	1.9	1
6	0.41	0.04	−0.5	1.7
17	−0.29	0.04	0.3	1.7
21	0.59	0.05	0.9	0.9
8	−0.24	0.04	−0.9	−0.4
12	0.37	0.04	−1.6	−0.4
16	−0.44	0.05	−1.3	−1.1
7	0.38	0.04	−2.1	−2.2
20	−0.29	0.04	−3.5	−3.8
19	−0.25	0.04	−3.7	−3.7
2	−0.36	0.04	−3.9	−4.1
14	−0.24	0.04	−4.1	−4.1
13	0.5	0.05	−4.6	−4.5
3	−0.19	0.04	−7.2	−6.9
4	0.01	0.04	−7.7	−7.2

**Table 4:** Total and subscale scores.

Subscales	Sex				*p*	Region				*p*	Specialization				*p*	Private-state praxis				*p*
	Men		Women			Other		Athens			Specialists		Residents			Private		State		
	Mean	SD	Mean	SD		Mean	SD	Mean	SD		Mean	SD	Mean	SD		Mean	SD	Mean	SD	
Fundamentals Subscale	14.11	3.86	13.35	3.83	**0.011**	14.24	3.73	13.15	3.99	**0.004**	13.91	4.15	13.66	3.24	0.476	13.90	4.18	13.72	3.31	0.383
State Audit Subscale	10.64	3.61	10.51	3.25	0.690	10.48	3.38	10.77	3.63	0.385	10.65	3.69	10.47	3.03	0.593	10.76	3.72	10.31	3.04	**0.001**
Empathy Subscale	11.05	3.56	9.86	2.98	**<0.001**	10.99	3.47	9.98	3.19	**0.002**	10.90	3.45	10.04	3.23	**0.009**	10.84	3.43	10.23	3.31	0.907
Fiscal Subscale	18.31	4.54	18.32	3.96	0.973	18.36	4.23	18.24	4.49	0.772	18.33	4.58	18.29	3.81	0.940	18.40	4.57	18.18	3.91	**0.010**
Physician Patient Interaction	5.83	1.91	5.64	1.79	0.287	5.91	1.83	5.52	1.90	**0.032**	5.78	1.93	5.72	1.74	0.762	5.80	1.94	5.69	1.74	**0.001**
Resources Utilization Subscale	6.75	2.26	6.37	2.26	0.084	6.84	2.18	6.23	2.35	**0.007**	6.69	2.35	6.45	2.09	0.262	6.65	2.37	6.55	2.10	0.766
Total score	66.69	14.67	64.06	12.90	**0.047**	66.81	13.90	63.89	14.22	**0.033**	66.26	15.04	64.64	12.00	0.213	66.35	15.09	64.68	12.28	**0.012**

Significance level was set to 0.05; higher values indicate better acceptance of generics.
